# Potential of non-contrast stress T1 mapping for the assessment of myocardial injury in hypertrophic cardiomyopathy

**DOI:** 10.1186/s12968-023-00966-5

**Published:** 2023-09-28

**Authors:** Hisanori Kosuge, Shoko Hachiya, Yasuhiro Fujita, Satoshi Hida, Taishiro Chikamori

**Affiliations:** Department of Cardiology, Tokyo Medical University, 6-7-1 Nishishinjuku, Shinjuku-ku, Tokyo, 160-0023 Japan

**Keywords:** Hypertrophic cardiomyopathy, T1 mapping, Myocardial injury, Coronary flow reserve

## Abstract

**Background:**

Ischemia of the hypertrophied myocardium due to microvascular dysfunction is related to a worse prognosis in hypertrophic cardiomyopathy (HCM). Stress and rest T1 mapping without contrast agents can be used to assess myocardial blood flow. Herein, we evaluated the potential of non-contrast stress T1 mapping in assessing myocardial injury in patients with HCM.

**Methods:**

Forty-five consecutive subjects (31 HCM patients and 14 control subjects) underwent cardiac magnetic resonance (CMR) at 3T, including cine imaging, T1 mapping at rest and during adenosine triphosphate (ATP) stress, late gadolinium enhancement (LGE), and phase-contrast (PC) cine imaging of coronary sinus flow at rest and during stress to assess coronary flow reserve (CFR). PC cine imaging was performed on 25 subjects (17 patients with HCM and 8 control subjects). Native T1 values at rest and during stress were measured using the 16-segment model, and T1 reactivity was defined as the change in T1 values from rest to stress.

**Results:**

ATP stress induced a significant increase in native T1 values in both the HCM and control groups (HCM: p < 0.001, control: p = 0.002). T1 reactivity in the HCM group was significantly lower than that in the control group (4.2 ± 0.3% vs. 5.6 ± 0.5%, p = 0.044). On univariate analysis, T1 reactivity correlated with native T1 values at rest, left ventricular mass index, and CFR. Multiple linear regression analysis demonstrated that only CFR was independently correlated with T1 reactivity (β = 0.449; 95% confidence interval, 0.048–0.932; p = 0.032). Furthermore, segmental analysis showed decreased T1 reactivity in the hypertrophied myocardium and the non-hypertrophied myocardium with LGE in the HCM group.

**Conclusions:**

T1 reactivity was lower in the hypertrophied myocardium and LGE-positive myocardium compared to non-injured myocardium. Non-contrast stress T1 mapping is a promising CMR method for assessing myocardial injury in patients with HCM.

*Trial registration* Retrospectively registered.

## Background

Hypertrophic cardiomyopathy (HCM) is a genetic cardiac disease characterized by inappropriate left ventricular hypertrophy [1]. Myocyte death and replacement fibrosis in HCM are caused by myocardial ischemia due to microvascular dysfunction [2].

Cardiac magnetic resonance (CMR) is a useful non-invasive imaging modality for the assessment of cardiac function and myocardial tissue characterization. Late gadolinium enhancement (LGE) can detect myocardial fibrosis, but it is ineffective for evaluating diffuse interstitial fibrosis [3]. T1 mapping, which includes native T1 values and extracellular volume (ECV), can identify myocardial edema and diffuse fibrosis. LGE and ECV are associated with adverse cardiac events in patients with HCM [4–6]. In the absence of obstructive coronary artery disease (CAD), coronary flow reserve (CFR) could be a marker of microvascular dysfunction [7]. CFR was quantified as the ratio of myocardial blood flow or coronary sinus flow after pharmacological stress to rest using positron emission tomography (PET) or CMR [7, 8]. CFR is impaired in patients with non-ischemic cardiomyopathy and heart failure with preserved ejection fraction [9–11]. An impaired CFR is an independent predictor of poor clinical outcomes in these patients [11].

It has been reported that adenosine stress and rest T1 mapping may be useful to distinguish between normal and ischemic myocardium in CAD without gadolinium contrast agents [12, 13]. The difference in T1 values between rest and adenosine stress (T1 reactivity) in infarcted and ischemic myocardium was significantly lower compared to normal myocardium. Additionally, patients with type 2 diabetes mellitus in the absence of CAD exhibited lower T1 reactivity compared to healthy controls, suggesting the involvement of microvascular dysfunction [14]. In contrast to CFR, T1 reactivity may enable the evaluation of myocardial injury globally and regionally because of the segmental measurement of T1 values.

In this study, we sought to evaluate the hypothesis that non-contrast T1 mapping at rest and during adenosine triphosphate (ATP) stress can detect myocardial injury in patients with HCM.

## Methods

### Study patients

We retrospectively enrolled 31 patients with HCM and 14 control subjects who underwent CMR imaging to evaluate suspected CAD between October 2018 and June 2022. In the HCM group, the electrocardiogram of all patients revealed ST-T changes including ST depression or negative T waves. Five patients presented with atypical chest discomfort and the remaining were asymptomatic. None of patients had a history of non-sustained ventricular tachycardia or syncope. HCM was diagnosed according to the JCS/JHFS guideline and was defined as left ventricular wall thickness ≥ 15 mm (in the absence of a family history of HCM), or 13–14 mm (with a family history of HCM) [15]. Patients with other cardiac diseases including storage, infiltrative, or systemic diseases were excluded [15]. Patients with stress perfusion defects underwent MR coronary angiography (MRCA) or computed tomography coronary angiography (CTCA). None of the patients had significant coronary artery stenosis. In the control subjects, 2 subjects presented with dyspnea on exertion and 12 presented with non-exertional chest discomfort. Subjects who exhibited no abnormalities on CMR imaging and had no history of cardiovascular diseases were included in the control group. Patients with known ischemic heart disease, more than moderate valvular heart disease, and contraindications for CMR were excluded from this study. This retrospective study of clinically acquired data was approved by the Institutional Review Board of Tokyo Medical University, and the need for written informed consent was waived.

### CMR protocol

**Fig. 1:**
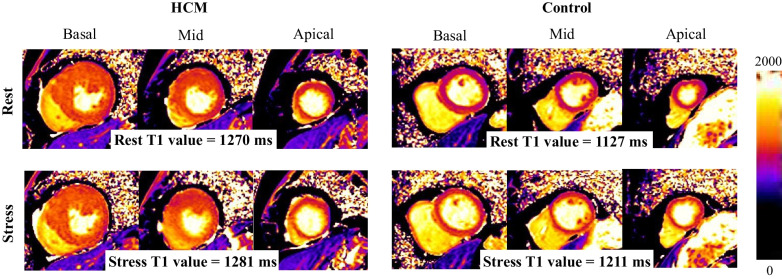
Representative images of T1 mapping at rest and during ATP stress

CMR imaging was performed using a Magnetom Skyra 3T system (Siemens Healthineers, Erlangen, Germany) with a 60-channel body coil. This included cine images, T1 mapping at rest and during ATP stress, late gadolinium enhancement (LGE), and phase-contrast (PC) cine imaging of coronary sinus flow at rest and during ATP stress to assess the coronary flow reserve (CFR). Patients were instructed not to consume caffeine for 12 h before ATP stress CMR. PC cine imaging was performed on 25 subjects (17 patients with HCM and 8 control subjects). Short-axis images covering the left ventricle (LV) from base to apex and 2-chamber, 3-chamber, and 4-chamber long-axis cine images were acquired with steady-state free precession to evaluate cardiac function and myocardial mass (repetition time (TR): 28.2 ms, echo time (TE): 1.6 ms, flip angle: 60°, field of view (FOV): 360 × 270 mm, acquisition matrix: 224 × 224, slice thickness: 6 mm, number of cardiac cycle phases: 30). T1 mapping at rest was performed on short-axis slices at the base, mid-ventricle, and apex (Fig. 1). A modified Look-Locker Inversion recovery (MOLLI) sequence with a 5(3)3 scheme was used (TR: 349 ms, TE: 1.1 ms, flip angle: 35°, FOV: 360 × 306 mm, acquisition matrix: 256 × 169, slice thickness: 8 mm, number of cardiac cycle phases: 1). Stress CMR was performed with a 3-min continuous intravenous injection of ATP (140 μg/kg/min). After a 3-min infusion, stress T1 mapping was performed on the same short-axis slices as ones of T1 mapping at rest using MOLLI sequence with a 5(3)3 scheme (Fig. 1). The MOLLI sequence was modified to reduce heart rate sensitivity [16, 17], although the reduction in heart rate sensitivity was insufficient compared to the shortened MOLLI sequence and the 5s(3s)3s-MOLLI sequence used in previous studies [12, 14, 18, 19]. Rest and stress PC cine imaging were performed immediately after rest and stress T1 mapping. The imaging plane of the PC cine images was positioned perpendicular to the coronary sinus 2 cm from the ostium [8]. PC cine images were acquired during shallow breath-holding (TR: 37.8 ms, TE: 2.6 ms, flip angle: 20°, FOV: 340 × 231 mm, acquisition matrix: 192 × 173, slice thickness: 6.0 mm, number of cardiac cycle phases: 40). Stress perfusion imaging was performed immediately after the stress PC cine imaging. Three short-axis slices identical to T1 mapping were obtained with breath-hold by intravenous administration of 0.1 mmol/kg of gadobutrol (Gadovist, Bayer Healthcare, Leverkusen, Germany) at a rate of 3 ml/s, followed by a 20 ml saline flush (TR: 410 ms, TE: 1.1 ms, flip angle: 35°, FOV: 360 × 306 mm, acquisition matrix: 256 × 169, slice thickness: 8 mm, number of cardiac cycle phases: 1), and ATP infusion was terminated. Ten minutes later, rest perfusion imaging was performed using the same protocol as that for stress perfusion. LGE images were obtained using an inversion-recovery gradient echo sequence 10 min after rest perfusion (TR: 700 ms, TE: 1.9 ms, flip angle: 12°, FOV: 360 × 270 mm, acquisition matrix: 192 × 157, slice thickness: 6 mm, number of cardiac cycle phases: 1). The inversion time was adjusted to null normal myocardium for each patient.

### Image analysis

CMR images were analyzed in a blinded fashion by two observers using commercially available CMR postprocessing software (Ziostation 2, Ziosoft, Tokyo, Japan). Left ventricular volume, ejection fraction, and mass were calculated by manual tracing of endocardial and epicardial contours of the end-diastolic and end-systolic short-axis cine images [20]. Perfusion imaging and T1 mapping were performed using a 16-segment model. The presence of a perfusion defect was defined by hypoenhancement in each segment that persisted for at least 3 consecutive frames after maximal myocardial enhancement [9, 21]. A total of 626 segments for T1 reactivity and 689 segments for native T1 values from 45 patients were available for analysis. The segments in the HCM group were classified as hypertrophied and non-hypertrophied myocardial segments. Hypertrophy was defined as left ventricular wall thickness of ≥ 13 mm. Native T1 values were measured by placing a region of interest in each segment, and T1 reactivity was calculated as follows: (T1stress − T1rest)/T1rest × 100 (%). Each region of interest was carefully drawn avoiding the adjacent blood pool and epicardium. The numbers of segments for T1 reactivity and native T1 were different because there were more segments after stress that could not be measured as T1 values, relative to the segments at rest, due to insufficient image quality. Segments with or without LGE in the HCM group were visually assessed. Blood flow in the coronary sinus was measured by tracing the contour of the coronary sinus on the magnitude images in each frame. Coronary sinus flow (CSF) was corrected using the rate pressure product as follows: rate pressure product = systolic blood pressure × heart rate, corrected CSF = CSF/rate pressure product × 7500, and CFR was calculated as corrected CSF during stress divided by corrected CSF at rest [7, 20]. Intraobserver and interobserver agreements of native T1 measurements from a random sample of 10 subjects were assessed by two independent observers. Interstudy reproducibility was assessed in three subjects.

### Statistical analysis

All data are expressed as a mean ± standard error of the mean. A chi-square analysis was performed to evaluate the association between the collected clinical information. The Mann–Whitney U test was used to compare age, body mass index (BMI), and CMR data between the HCM and control groups. This test was also used to compare native T1 values at rest (rest T1 values) and T1 reactivity between LGE-positive and LGE-negative groups and segments. The difference in T1 reactivity between the three groups was calculated using a 1-way analysis of variance with Bonferroni’s post hoc test. The Kruskal–Wallis test was used to compare resting T1 values between the three and four groups and T1 reactivity among the four groups, followed by Dunn’s post hoc test with Bonferroni correction. Univariable and multivariable linear regression analyses were used to evaluate the relationship between T1 reactivity and the clinical and imaging parameters. All variables associated with T1 reactivity (p < 0.2) were included in multivariable linear regression analysis [22]. Area under the curve (AUC) was calculated to assess the comparison between T1 reactivity and myocardial perfusion defect. Intraobserver and interobserver agreements of native T1 measurements were assessed with intraclass correlation coefficients. The Shapiro–Wilk test was used to assess normality for each variable. All analyses were performed using the SPSS software (SPSS 26.0, Chicago, IL, USA). Statistical significance was set at p-value < 0.05.

## Results

### Patient characteristics

The clinical characteristics of the patients are summarized in Table 1. There were no significant differences in age, sex, BMI, smoking frequency, hypertension, diabetes mellitus, or hyperlipidemia between the HCM and control groups. The HCM group included six patients with apical hypertrophy and two patients with left ventricular outflow obstruction; aneurysm formation was not seen in this group.

**Table 1:** Patient characteristics

Characteristic	HCM (n = 31)	Control (n = 14)	p value
Age (years)	62.3 ± 2.6	58.6 ± 4.3	0.677
Male n (%)	25 (80.6%)	10 (71.4%)	0.491
BMI (kg/m^2^)	23.8 ± 0.60	22.2 ± 0.55	0.100
Smoking	18 (58.1%)	4 (28.6%)	0.067
Hypertension	17 (54.8%)	6 (42.9%)	0.457
Diabetes mellitus	4 (12.9%)	1 (7.1%)	0.569
Hyperlipidemia	9 (29.0%)	3 (21.4%)	0.593

Data are mean ± standard deviation, or number with percentage

*BMI* body mass index

### CMR findings

The CMR data are listed in Table 2. There were no significant differences in stress and resting heart rate, left ventricular ejection fraction (LVEF), and LV volumes between the HCM and control groups. The LV mass index (LVMI) was significantly greater in the HCM group compared to the control group (60.5 ± 3.4 g/m^2^ vs. 39.6 ± 1.6 g/m^2^, p < 0.001). There were no statistically significant differences in systolic and diastolic blood pressures between the HCM and control groups (systolic pressure: 137 ± 3 mmHg vs. 144 ± 11 mmHg, p = 0.923, diastolic pressure: 85 ± 2 mmHg vs. 83 ± 7 mmHg, p = 0.468).

**Table 2:** Cardiac magnetic resonance findings

Variables	HCM (n = 31)	Control (n = 14)	p value
LVEF (%)	54.6 ± 1.9	55.3 ± 1.5	0.507
EDV (ml)	127.0 ± 6.7	111.8 ± 6.1	0.292
EDVI (ml/m^2^)	73.8 ± 3.5	69.1 ± 3.6	0.573
ESV (ml)	60.2 ± 5.8	52.0 ± 4.4	0.806
ESVI (ml/m^2^)	34.9 ± 3.0	32.0 ± 2.6	0.902
LV mass (g)	104.5 ± 6.5	64.2 ± 2.9	< 0.001
LV mass index (g/m^2^)	60.5 ± 3.4	39.6 ± 1.6	< 0.001
Resting heart rate (bpm)	69 ± 2	70 ± 3	0.778
The presence of LGE	23 (74.2%)	0 (0%)	< 0.001
Rest T1 (ms)	1244 ± 9	1197 ± 10	0.004
Stress T1 (ms)	1285 ± 10	1248 ± 10	0.02
T1 reactivity (%)	4.24 ± 0.34	5.57 ± 0.49	0.044

Data are mean ± standard deviation, or number with percentage

*LVEF* left ventricular ejection fraction; *EDV* end-diastolic volume; *EDVI* end-diastolic volume index; *ESV* end-systolic volume; *ESVI* end-systolic volume index; *LGE* late gadolinium enhancement

### Native T1 values and T1 reactivity

**Fig. 2:**
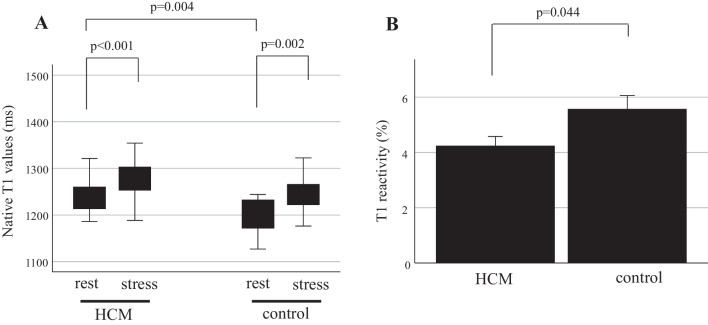
Native T1 values at rest and during ATP stress (**A**) and T1 reactivity (**B**) in patients with HCM and control subjects

**Fig. 3:**
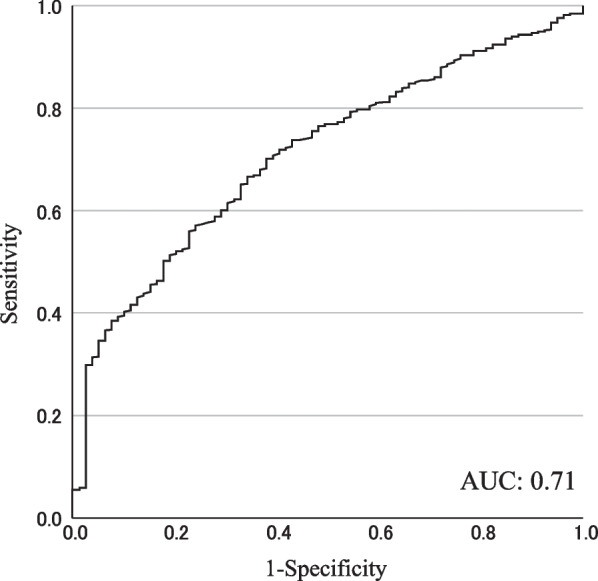
The receiver operating characteristic curve for T1 reactivity to detect myocardial perfusion defects

Resting T1 values in the HCM group were significantly higher compared to those in the control group (1244 ± 9 ms vs. 1197 ± 10 ms, p = 0.004, Fig. 1A). Native T1 values during stress (stress T1 values) significantly increased in both the HCM and control groups compared to those at rest (HCM: 1285 ± 10 ms, p < 0.001 vs. rest, control: 1248 ± 10 ms, p = 0.002 vs. rest, Fig. 2A). T1 reactivity in the HCM group was significantly lower compared to the control group (4.2 ± 0.3% vs. 5.6 ± 0.5%, p = 0.044, Fig. 2B). Nineteen percent of segments in the HCM group showed myocardial perfusion defect, while there were no segments positive for perfusion defect in the control group. T1 reactivity showed an AUC of 0.71 (95% confidence interval (CI), 0.66–0.77) for detecting myocardial perfusion defects; the cut-off value of T1 reactivity was 4.3% (Fig. 3). Using this cut-off value, sensitivity and specificity to differentiate myocardial injury was 56% and 77%, respectively. The interclass correlation coefficient for intraobserver agreements of native T1 (rest T1 and stress T1) measurements were 0.990 (95% CI 0.963–0.997) and 0.993 (95% CI 0.974–0.998). The interclass correlation coefficient for interobserver agreements of rest T1 and stress T1 measurements were 0.984 (95% CI 0.936–0.996) and 0.996 (95% CI 0.985–0.999). Interstudy reproducibility was assessed in three subjects. Mean interval between the first and second scans was 16.3 ± 5.2 months. There was no change in clinical findings between the first and second scans. T1 reactivity of the first scan was not significantly different compared to that of the second scan (4.3 ± 1.0% vs. 4.2 ± 0.8%, p = 0.474) and there was no significant bias on the Bland–Altman analysis (0.14 ± 0.27%).

In HCM patients, resting T1 values and T1 reactivity showed no significant differences between LGE-positive and LGE-negative groups (resting T1: 1248 ± 11 ms vs. 1232 ± 11 ms, p = 0.464, T1 reactivity: 4.0 ± 0.4% vs. 5.0 ± 0.7%, p = 0.203). With regards segmental analysis, segments with LGE showed significantly higher rest T1 values and lower T1 reactivity than those without LGE (resting T1: 1300 ± 9 ms vs. 1235 ± 3 ms, p < 0.001, T1 reactivity: 3.4 ± 0.4% vs. 4.9 ± 0.2%, p < 0.001).

### Relationship between T1 reactivity and the clinical and imaging parameters

CFR was significantly lower in the HCM group compared to the control group (2.0 ± 0.3 vs. 4.2 ± 0.8, p = 0.003). Univariate analysis showed that resting T1 values, LVMI, and CFR were correlated with T1 reactivity. However, there was no significant correlation between T1 reactivity and age, BMI, or LVEF. Multiple linear regression analysis demonstrated that only CFR was independently correlated with T1 reactivity (β = 0.449, p = 0.032; Table 3), but no independent correlation was found between T1 reactivity, and rest T1 values and LVMI.

**Table 3:** Univariate and multivariate analysis of relationship between T1 reactivity and clinical and CMR findings

	Multivariate			Univariate		
	β	95% CI for B	p value	β	95% CI for B	p value
Age	0.21	(− 0.012, 0.068)	0.166	0.064	(− 0.054, 0.072)	0.762
BMI (kg/m^2^)	− 0.02	(− 0.210, 0.183)	0.889			
LVEF (%)	− 0.06	(− 0.077, 0.053)	0.708			
Rest T1 (ms)	− 0.37	(− 0.025, − 0.003)	0.013	− 0.14	(− 0.027, 0.014)	0.524
Coronary flow reserve	0.552	(0.210, 0.995)	0.004	0.449	(0.048, 0.932)	0.032
LV mass index (g/m^2^)	− 0.43	(− 0.075, − 0.017)	0.003	− 0.17	(− 0.067, 0.035)	0.524

*BMI* body mass index; *LVEF* left ventricular ejection fraction

### Comparison of rest T1 values and T1 reactivity between hypertrophied and non-hypertrophied myocardium

**Fig. 4:**
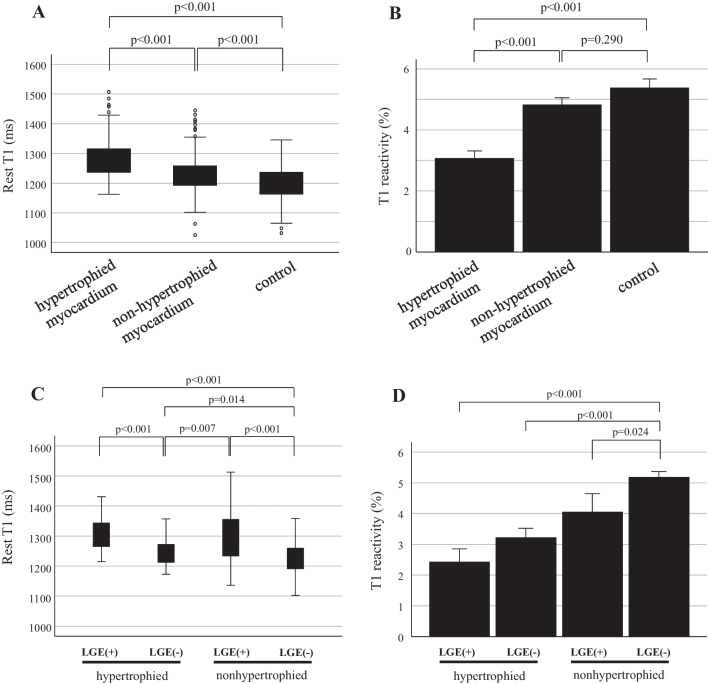
Rest T1 (**A**) and T1 reactivity (**B**) in hypertrophied and non-hypertrophied myocardium in patients with HCM and control myocardium. Rest T1 (**C**) and T1 reactivity (**D**) in hypertrophied and non-hypertrophied myocardium with or without LGE in patients with HCM and control myocardium

Resting T1 values in the hypertrophied myocardium in the HCM group (143 segments: 1280 ± 5.6 ms) were significantly higher compared to those in the non-hypertrophied myocardium in the HCM (335 segments: 1229 ± 3.4 ms, p < 0.001, Fig. 4A) and control (211 segments: 1198 ± 3.8 ms, p < 0.001, Fig. 4A) groups. Furthermore, resting T1 values in the non-hypertrophied myocardium in the HCM group was significantly higher compared to those in the control group (p < 0.001, Fig. 4A). T1 reactivity in the hypertrophied myocardium in the HCM group (139 segments: 3.1 ± 0.2%) was significantly lower than that in the non-hypertrophied myocardium in the HCM (301 segments: 4.8 ± 0.2%, p < 0.001, Fig. 4B) and control (169 segments: 5.4 ± 0.3%, p < 0.001, Fig. 4B) groups. However, there was no statistically significant difference in T1 reactivity between the non-hypertrophied myocardium in the HCM and control groups (p = 0.290, Fig. 4B).

In the HCM group, the hypertrophied and non-hypertrophied myocardium with LGE had significantly higher resting T1 values compared to those without LGE (p < 0.001). Resting T1 values in the hypertrophied myocardium without LGE were significantly higher compared to those in the non-hypertrophied myocardium without LGE (p = 0.014). There was no statistically significant difference in the rest T1 values between the hypertrophied myocardium with LGE and the non-hypertrophied myocardium with LGE (Fig. 4C). The non-hypertrophied myocardium without LGE revealed significantly higher T1 reactivity than the hypertrophied myocardium with and without LGE (p < 0.001) and the non-hypertrophied myocardium with LGE (p = 0.024). There were no statistically significant differences among the three groups (Fig. 4D).

## Discussion

This study showed that patients with HCM had a higher resting T1 values and lower T1 reactivity compared to control subjects, and T1 reactivity was correlated only with CFR measured by PC cine MRI. Additionally, T1 reactivity was significantly lower in the hypertrophied myocardium of the HCM group compared to the non-hypertrophied myocardium of the HCM and control groups. T1 reactivity in the hypertrophied and non-hypertrophied myocardium with LGE was equivalent to that in the hypertrophied myocardium without LGE. To the best of our knowledge, this study is the first to show that T1 reactivity can assess myocardial injury globally and regionally in HCM patients without a gadolinium contrast agent.

Stress perfusion imaging with MRI and PET has been reported to be useful for the detection of hemodynamically significant CAD and the determination of invasive revascularization [23, 24]. CFR is defined as the ratio of hyperemic to resting myocardial blood flow and can estimate abnormalities in the structure and function of coronary macrocirculation and microcirculation [25]. CFR can be assessed noninvasively by PC cine MRI and PET and shows the prognostic value and risk stratification for patients with known or suspected CAD [26, 27]. Furthermore, in the absence of obstructive epicardial CAD, reduced CFR indicates coronary microvascular dysfunction [11]. Microvascular dysfunction portends adverse cardiovascular events in patients without obstructive CAD and with non-ischemic cardiomyopathy and heart failure with preserved ejection fraction [7, 10, 11, 28, 29]. In HCM, microvascular dysfunction may cause cardiomyocyte necrosis due to ischemia, followed by replacement myocardial fibrosis. Myocardial fibrosis was found to increase in proportion to LV wall thickness [30]. Myocardial blood flow response to pharmacological stress assessed by PET and MRI showed a blunt response in the HCM group compared to the control group. Myocardial perfusion reserve (MPR) measured by MRI correlates well with PET in healthy humans [31], but MRI is advantageous for the measurement of MPR due to the absence of radiation exposure. However, there are few reports regarding the measurement of CFR using PC cine MRI in HCM. In this study, the CFR measured by PC cine MRI was significantly lower in patients with HCM compared to controls.

T1 mapping can be used for myocardial tissue characterization, and patients with HCM showed significantly higher native T1 values and ECV compared to healthy volunteers despite the presence of LGE [32]. Furthermore, stress T1 mapping may be useful for the differentiation of myocardial tissue classes and assessment of coronary vasoreactivity [12–14, 33]. In the absence of obstructive epicardial CAD, the degree of coronary vasoreactivity represented as T1 reactivity may be associated with the function of coronary microcirculation; however, the association between T1 reactivity and the function of coronary microcirculation has not been fully investigated. In this study, we demonstrated that T1 reactivity was significantly lower in the HCM group than in the control group and that CFR, which may represent microvascular dysfunction, was independently correlated with T1 reactivity. Additionally, in contrast to CFR, T1 reactivity may be able to regionally evaluate the function of coronary microcirculation by segmental analysis. Segmental analysis showed that T1 reactivity was significantly lower in the hypertrophied myocardium in the HCM group compared to control segments, but there was no statistically significant difference in T1 reactivity between the non-hypertrophied myocardium in the HCM and control groups. Hypertrophied and non-hypertrophied myocardium in the HCM group showed significantly higher resting T1 values compared to the control group, which was in line with the results of Huang et al. [34]. These results suggest the involvement of microvascular dysfunction in the hypertrophied myocardium of the HCM group. The findings observed in the nonhypertrophied myocardium may contribute to the preserved vasodilation response aside from interstitial expansion, as reported by Mahmod et al. [18]. However, Camici et al. reported that microvascular dysfunction was observed in hypertrophied and non-hypertrophied myocardium in patients with HCM using PET [35]. In that study, the analysis of blood flow was performed only on the interventricular septum and LV free wall, which was different from ours using a 16-segmental model. The discrepancy in results may be associated with differences in the analysis methods.

Autopsy in patients with heart failure with preserved ejection fraction revealed that microvascular density was correlated with the severity of myocardial fibrosis [36]. Ma et al. reported that T1 reactivity showed positive high correlation with microvascular density and negative moderate correlation with collagen volume fraction measured by histology in a rabbit model of type 2 diabetes mellitus, indicating that impaired microvascular function is associated with myocardial injury [37]. Furthermore, T1 reactivity is useful to assess myocardial injury by differentiating myocardial tissue characteristics without a gadolinium contrast agent and can be also available for the patient with severe chronic kidney disease [12, 13]. In the current study, T1 reactivity did not show statistically significant differences among the hypertrophied myocardium with and without LGE and the non-hypertrophied myocardium with LGE, although the hypertrophied myocardium without LGE showed significantly lower rest T1 values than the hypertrophied myocardium and non-hypertrophied myocardium with LGE (Fig. 4C, D) Therefore, changes in T1 reactivity may be explained by myocardial injury induced by perfusion abnormalities.

### Limitations

This study has some limitations. First, the sample size was small. Therefore, a larger prospective study is required to confirm these findings. Second, MRCA or CTCA was performed to evaluate CAD in subjects with positive stress perfusion. Patients with significant CAD were excluded, but invasive coronary angiography was not performed. Therefore, the exclusion of significant CAD may have been incomplete in this study. Third, the perfusion defect was assessed visually and the diagnostic accuracy for detecting myocardial perfusion defect by T1 reactivity was moderate. The measurement of myocardial blood flow or myocardial perfusion reserve is warranted in future investigations to examine the comparison between T1 reactivity and myocardial hypoperfusion. Finally, 13.1% of myocardial segments were excluded because of insufficient image quality during ATP stress. The MOLLI sequence used in this study was modified to reduce heart rate sensitivity [16, 17], but the reduction in heart rate sensitivity was insufficient compared to the shortened MOLLI sequence and the 5s(3s)3s-MOLLI sequence used in previous studies [12, 14, 18, 19]. However, the proportion of myocardial segments with insufficient image quality in this study was comparable to that reported in previous studies. Therefore, further investigation is needed to improve diagnostic performance.

## Conclusions

In conclusion, T1 reactivity was decreased in hypertrophied myocardium and LGE-positive myocardium compared with non-injured myocardium. Our results suggest the potential use of non-contrast stress T1 mapping for evaluating myocardial injury globally and regionally in patients with HCM; however, further studies are needed to elucidate the clinical significance of these findings.

## Data Availability

The datasets used and/or analyzed during the current study are available from the corresponding author upon reasonable request.

## Abbreviations

HCM: Hypertrophic cardiomyopathy
CMR: Cardiac magnetic resonance
LGE: Late gadolinium enhancement
ECV: Extracellular volume
CAD: Coronary artery disease
CFR: Coronary flow reserve
PET: Positron emission tomography
ATP: Adenosine triphosphate
PC: Phase-contrast
MOLLI: Modified Look-Locker Inversion recovery
CSF: Coronary sinus flow
BMI: Body mass index
AUC: Area under the curve
LVEF: Left ventricular ejection fraction
LVMI: Left ventricular mass index
CI: Confidence interval
MRI: Magnetic resonance imaging
MPR: Myocardial perfusion reserve

## References

[1] Marian AJ, Braunwald E (2017). Hypertrophic cardiomyopathy: genetics, pathogenesis, clinical manifestations, diagnosis, and therapy. Circ Res.

[2] Crea F, Camici PG, Bairey Merz CN (2014). Coronary microvascular dysfunction: an update. Eur Heart J.

[3] Ambale-Venkatesh B, Lima JA (2015). Cardiac MRI: a central prognostic tool in myocardial fibrosis. Nat Rev Cardiol.

[4] Chan RH, Maron BJ, Olivotto I, Pencina MJ, Assenza GE, Haas T (2014). Prognostic value of quantitative contrast-enhanced cardiovascular magnetic resonance for the evaluation of sudden death risk in patients with hypertrophic cardiomyopathy. Circulation.

[5] Green JJ, Berger JS, Kramer CM, Salerno M (2012). Prognostic value of late gadolinium enhancement in clinical outcomes for hypertrophic cardiomyopathy. JACC Cardiovasc Imaging.

[6] Li Y, Liu X, Yang F, Wang J, Xu Y, Fang T (2021). Prognostic value of myocardial extracellular volume fraction evaluation based on cardiac magnetic resonance T1 mapping with T1 long and short in hypertrophic cardiomyopathy. Eur Radiol.

[7] Kato S, Saito N, Kirigaya H, Gyotoku D, Iinuma N, Kusakawa Y (2016). Impairment of coronary flow reserve evaluated by phase contrast cine-magnetic resonance imaging in patients with heart failure with preserved ejection fraction. J Am Heart Assoc.

[8] Cecchi F, Olivotto I, Gistri R, Lorenzoni R, Chiriatti G, Camici PG (2003). Coronary microvascular dysfunction and prognosis in hypertrophic cardiomyopathy. N Engl J Med.

[9] Kim EK, Lee SC, Chang SA, Jang SY, Kim SM, Park SJ (2020). Prevalence and clinical significance of cardiovascular magnetic resonance adenosine stress-induced myocardial perfusion defect in hypertrophic cardiomyopathy. J Cardiovasc Magn Reson.

[10] Gulati A, Ismail TF, Ali A, Hsu LY, Gonçalves C, Ismail NA (2019). Microvascular dysfunction in dilated cardiomyopathy: a quantitative stress perfusion cardiovascular magnetic resonance study. JACC Cardiovasc Imaging.

[11] Taqueti VR, Solomon SD, Shah AM, Desai AS, Groarke JD, Osborne MT (2018). Coronary microvascular dysfunction and future risk of heart failure with preserved ejection fraction. Eur Heart J.

[12] Liu A, Wijesurendra RS, Francis JM, Robson MD, Neubauer S, Piechnik SK (2016). Adenosine stress and rest T1 mapping can differentiate between ischemic, infarcted, remote, and normal myocardium without the need for gadolinium contrast agents. JACC Cardiovasc Imaging.

[13] Yimcharoen S, Zhang S, Kaolawanich Y, Tanapibunpon P, Krittayaphong R (2020). Clinical assessment of adenosine stress and rest cardiac magnetic resonance T1 mapping for detecting ischemic and infarcted myocardium. Sci Rep.

[14] Levelt E, Piechnik SK, Liu A, Wijesurendra RS, Mahmod M, Ariga R (2017). Adenosine stress CMR T1-mapping detects early microvascular dysfunction in patients with type 2 diabetes mellitus without obstructive coronary artery disease. J Cardiovasc Magn Reson.

[15] Kitaoka H, Tsutsui H, Kubo T, Ide T, Chikamori T, Fukuda K (2021). JCS/JHFS 2018 guideline on the diagnosis and treatment of cardiomyopathies. Circ J.

[16] Kellman P, Wilson JR, Xue H, Ugander M, Arai AE (2012). Extracellular volume fraction mapping in the myocardium, part 1: evaluation of an automated method. J Cardiovasc Magn Reson.

[17] Piechnik SK, Neubauer S, Ferreira VM (2018). State-of-the-art review: stress T1 mapping-technical considerations, pitfalls and emerging clinical applications. MAGMA.

[18] Mahmod M, Piechnik SK, Levelt E, Ferreira VM, Francis JM, Lewis A (2014). Adenosine stress native T1 mapping in severe aortic stenosis: evidence for a role of the intravascular compartment on myocardial T1 values. J Cardiovasc Magn Reson.

[19] Bohnen S, Prüßner L, Vettorazzi E, Radunski UK, Tahir E, Schneider J (2019). Stress T1-mapping cardiovascular magnetic resonance imaging and inducible myocardial ischemia. Clin Res Cardiol.

[20] Tezuka D, Kosuge H, Terashima M, Koyama N, Kishida T, Tada Y (2018). Myocardial perfusion reserve quantified by cardiac magnetic resonance imaging is associated with late gadolinium enhancement in hypertrophic cardiomyopathy. Heart Vessels.

[21] Nakamori S, Sakuma H, Dohi K, Ishida M, Tanigawa T, Yamada A (2018). Combined assessment of stress myocardial perfusion cardiovascular magnetic resonance and flow measurement in the coronary sinus improves prediction of functionally significant coronary stenosis determined by fractional flow reserve in multivessel disease. J Am Heart Assoc.

[22] Zemek R, Barrowman N, Freedman SB, Gravel J, Gagnon I, McGahern C (2016). Clinical risk score for persistent postconcussion symptoms among children with acute concussion in the ED. JAMA.

[23] Takx RA, Blomberg BA, El Aidi H, Habets J, de Jong PA, Nagel E (2015). Diagnostic accuracy of stress myocardial perfusion imaging compared to invasive coronary angiography with fractional flow reserve meta-analysis. Circ Cardiovasc Imaging.

[24] Nagel E, Greenwood JP, McCann GP, Bettencourt N, Shah AM, Hussain ST (2019). Magnetic resonance perfusion or fractional flow reserve in coronary disease. N Engl J Med.

[25] Taqueti VR, Di Carli MF (2018). Coronary microvascular disease pathogenic mechanisms and therapeutic options: JACC state-of-the-art review. J Am Coll Cardiol.

[26] Murthy VL, Naya M, Foster CR, Hainer J, Gaber M, Di Carli G (2011). Improved cardiac risk assessment with noninvasive measures of coronary flow reserve. Circulation.

[27] Kato S, Saito N, Nakachi T, Fukui K, Iwasawa T, Taguri M (2017). Stress perfusion coronary flow reserve versus cardiac magnetic resonance for known or suspected cad. J Am Coll Cardiol.

[28] Taqueti VR, Hachamovitch R, Murthy VL, Naya M, Foster CR, Hainer J (2015). Global coronary flow reserve is associated with adverse cardiovascular events independently of luminal angiographic severity and modifies the effect of early revascularization. Circulation.

[29] Ford TJ, Ong P, Sechtem U, Beltrame J, Camici PG, Crea F (2020). Assessment of vascular dysfunction in patients without obstructive coronary artery disease: why, how, and when. JACC Cardiovasc Interv.

[30] Petersen SE, Jerosch-Herold M, Hudsmith LE, Robson MD, Francis JM, Doll HA (2007). Evidence for microvascular dysfunction in hypertrophic cardiomyopathy: new insights from multiparametric magnetic resonance imaging. Circulation.

[31] Fritz-Hansen T, Hove JD, Kofoed KF, Kelbaek H, Larsson HB (2008). Quantification of MRI measured myocardial perfusion reserve in healthy humans: a comparison with positron emission tomography. J Magn Reson Imaging.

[32] Xu J, Zhuang B, Sirajuddin A, Li S, Huang J, Yin G (2020). MRI T1 mapping in hypertrophic cardiomyopathy: evaluation in patients without late gadolinium enhancement and hemodynamic obstruction. Radiology.

[33] Burrage MK, Shanmuganathan M, Masi A, Hann E, Zhang Q, Popescu IA (2021). Cardiovascular magnetic resonance stress and rest T1-mapping using regadenoson for detection of ischemic heart disease compared to healthy controls. Int J Cardiol.

[34] Huang L, Ran L, Zhao P, Tang D, Han R, Ai T (2019). MRI native T1 and T2 mapping of myocardial segments in hypertrophic cardiomyopathy: tissue remodeling manifested prior to structure changes. Br J Radiol.

[35] Camici P, Chiriatti G, Lorenzoni R, Bellina RC, Gistri R, Italiani G (1991). Coronary vasodilation is impaired in both hypertrophied and nonhypertrophied myocardium of patients with hypertrophic cardiomyopathy: a study with nitrogen-13 ammonia and positron emission tomography. J Am Coll Cardiol.

[36] Mohammed SF, Hussain S, Mirzoyev SA, Edwards WD, Maleszewski JJ, Redfield MM (2015). Coronary microvascular rarefaction and myocardial fibrosis in heart failure with preserved ejection fraction. Circulation.

[37] Ma P, Liu J, Hu Y, Chen L, Liang H, Zhou X, Shang Y, Wang J (2023). Stress CMR T1-mapping technique for assessment of coronary microvascular dysfunction in a rabbit model of type II diabetes mellitus: validation against histopathologic changes. Front Cardiovasc Med.

